# Clinical Lectures on Diseases Peculiar to Women

**Published:** 1873-04

**Authors:** 


*Clinical Lectures on Diseases Peculiar to Women.* By Lombe Atthill, M. D., Univ. Dub., etc. Second Edition. Revised and enlarged, with six Lithographic Plates and Wood Cut illustrations. Philadelphia: Lindsay & Blackiston, 1873. Buffalo: T. Butler & Son.

This work is the substance of lectures delivered to the classes at Adelaide Hospital. It does not pretend to embrace as much as the larger and more extended treatises, but is a simple record of the observations and views of the author, which will, as a general thing, be found to agree with those of the profession. We can hardly agree with him in the frequent use of the pessary or so-called mechanical treatment, but with this exception his book will be found worthy of study.

